# Continuous high-soy protein soymilk intake affects ordinary walking speed in the Japanese pre-frail and frail elderly: a randomized controlled trial

**DOI:** 10.1186/s12877-024-05539-4

**Published:** 2025-01-15

**Authors:** Nene Sato, Yuji Terashima, Makoto Sugawara, Ryoichi Unno, Hiroaki Asao, Mitsuhiro Iwasaki, Tomoyuki Watanabe, Tomoko Uno, Mitsuo Maruyama

**Affiliations:** 1 Research and Development Division, MARUSAN-AI Co., Ltd., Okazaki, Aichi 444-2193 Japan; 2 Department of Health and Nutritional Sciences, Faculty of Health Sciences, Aichi Gakuin University, Nisshin, Japan; 3 Geroscience Research Center, Research Institute, National Center for Geriatrics and Gerontology, Obu, Aichi 474-8511 Japan; 4 Department of Aging Research, Nagoya University Graduate School of Medicine, Nagoya, Japan

**Keywords:** Frailty, Soy protein, Soymilk, Nutritional intervention, Walking speed

## Abstract

**Background:**

To investigate whether continuous intervention using soymilk containing high soy protein improves physical frailty, a randomized controlled trial was conducted among the Japanese pre-frail and frail elderly.

**Methods:**

Japanese pre-frail and frail elderly participants (*n* = 73) were randomly assigned to the high-soy protein and control groups, who then ingested soymilk containing 14.5 g/200 ml and 3.2 g/200 ml of soy protein, respectively. Before and after the 12-week intervention, walking speed, skeletal muscle mass, grip strength, and the revised Japanese CHS questionnaire regarding fatigue and physical activity were examined to evaluate the impact of each soymilk on physical frailty and compare the variation between the two groups. Physical activity (monitored using a pedometer), dietary intake (determined by questionnaire), and estimated protein intake (determined by casual urine testing) were also recorded before and after the intervention.

**Results:**

For the final analysis of the entire cohort (*n* = 70), there were no significant differences in the endpoints between the two groups. In the subgroup analysis, among participants with a walking speed of at least 1 m/s (*n* = 35, *P* = 0.012) and at least 5,000 steps/day before intervention (*n* = 27, *P* = 0.0083), the variation in walking speed after the 12-week intervention was significantly higher in the high-soy protein group than in the control group. Estimated protein intake was also significantly higher in the high-soy protein group than in the control group after the intervention. Regarding physical activity and dietary intake, no significant differences were observed between the groups before or after the intervention.

**Conclusion:**

The continuous 12-week intervention of high soy protein increased the walking speed among the Japanese pre-frail and frail elderly participants who had an ordinarily high walking speed and high step counts.

**Trial registration:**

UMIN Clinical Trials Registry, UMIN000044999. Registered July 29, 2021; https://center6.umin.ac.jp/cgi-open-bin/ctr/ctr_view.cgi?recptno=R000051409.

## Background

In nursing care, not only the prevention of age-related diseases is important but also the prevention of frailty [1]. Frailty encompasses physical frailty, such as sarcopenia and locomotive syndrome, psychological frailty, as in cognitive impairment and depression, and social frailty, characterized as being withdrawn and living alone [2]. Among them, physical frailty is particularly influenced by nutritional status [2]. Several cohort studies have considered the impacts of nutrition on frailty. For instance, a large national or international epidemiological study, known as the ABC Study [3] or the Kameoka Study [4], revealed that the higher the daily protein intake, the lower the risk of frailty. Protein intake generally promotes muscle synthesis, which may prevent the age-related reduction in muscle strength and mass, referred to as sarcopenia [5]. Various randomized controlled trials (RCTs) have concluded that nutritional interventions based on protein and amino acid intake, combined with exercise interventions, are beneficial for improving physical function [6–8]. However, few reports of RCTs showing an improvement in physical activity among the elderly with frailty or sarcopenia by nutritional intervention alone have mentioned that exercise intervention should be combined with nutritional intervention [9].

Previous RCTs have been performed in England using vegetable soy protein, showing that nutritional intervention alone is not sufficient for the frail elderly, but the combination with exercise is efficacious [10]. Conversely, some studies have found that soy protein intake improves locomotor function in the elderly with sarcopenia [11], as well as muscle strength and mass in low-activity participants [12]. Thus, the impact of soy protein on physical frailty remains unclarified.

In this study, we investigated the impact of a continuous nutritional intervention using soy protein on physical frailty. Specifically, we conducted an RCT based on the hypothesis that the frail or pre-frail status would improve in Japanese elderly participants if they ingested soymilk with a high soy protein content (14.5 g protein/200 ml) for 12 weeks, compared with similar participants who ingested soymilk with an ordinary soy protein content (3.2 g protein/200 ml) as the control.

## Methods

In this 12-week randomized, double-blind, parallel-group study, a total of 105 adults aged 65 to 85 years were screened for at least one of the criteria from the revised Japanese version of the Cardiovascular Health Study (hereinafter referred to as J-CHS criteria) [13]. As a randomized trial, the CONSORT 2010 checklist criteria was also used.

Subjects were recruited as paid volunteers between May 2, 2022 and June 27, 2022 by Macromill, Inc. (Tokyo), ASMARQ Co., Ltd. (Tokyo), and Nambu Co., Ltd. (Aichi). The sample size was calculated using G*Power software with an effect size of 0.8, an alpha error of 0.05, and a power of 0.80 for unpaired two-group tests. This yielded a required number per group of 26. To account for dropouts and subgroup analysis, the desired number of subjects per group was set at 45.

The following exclusion criteria were used: allergy to the soy food, daily high intake of soy food, regular use of drugs or health supplements that may influence the study results, and participation in other interventional trials one month prior to this study, or scheduled for after this study.

The screening results indicated that 73 of the 105 subjects were eligible. The target number of subjects was set at 90, but due to the impact of the spread of COVID-19, the number of eligible subjects was limited to 73. However, since the required number of 26 subjects per group was met, the study was started at the discretion of the principal investigator. Once all the available subjects had been recruited, they were stratified by walking speed and median age at the pre-intake test and randomly assigned to the high-soy protein group (*n* = 36) and the control group (*n* = 37). The principal investigator was responsible for subject enrollment, and the allocation was performed by the researcher responsible for randomization.

Subjects in the high-soy protein group received soymilk containing 14.5 g/200 ml of soy protein (160 kcal of energy, 14.5 g of protein, 9.3 g of fat, 4.9 g of carbohydrates, 0.8 g of fiber), and in the control group, subjects received soymilk containing 3.2 g/200 ml of soy protein (137 kcal of energy, 3.2 g of protein, 2.1 g of fat, 26.7 g of carbohydrates, and 0.6 g of fiber) for 12 weeks. The time of day when the soymilk was ingested was not specified.

The test and control foods were provided in plain packaging to prevent any potential bias due to their appearance. All relevant parties were blinded, including subjects, interventionists, outcome assessors, and the principal investigator. The researcher responsible for allocation created a correspondence table between the allocation information and subject information, and another person not involved in the study kept this table securely concealed until the data analysis was completed, thereby maintaining blinding.

The following outcomes and survey items were measured before and after the 12 weeks of intervention. The primary outcome was the change in walking speed after the intervention. Walking speed was determined based on the time required to walk on a 5-meter walking path with a 1-meter walk-in period.

The secondary outcomes were the change in skeletal muscle mass, grip strength, and J-CHS criteria questionnaire items (fatigue and physical activity) after the intervention. In addition, cognitive status was evaluated based on the change in Hasegawa’s Dementia Scale-Revised (HDS-R) [14]. Skeletal muscle mass and grip strength in the dominant hand were measured using an InBody570 instrument (InBody Co., Ltd.) and a digital grip strength meter (T.K.K. 5401, Takei Scientific Instruments Co., Ltd.), respectively.

Other analysis items included dietary intake, estimated protein intake, and physical activity before and after the intervention. Dietary intake was surveyed using the brief-type self-administered diet history questionnaire (BDHQ) [15]. Estimated protein intake was determined by measuring the urinary urea nitrogen and creatinine levels and calculated using the Tanaka [16] and Maloni formulas [17]. Physical activity and calorie consumption were monitored using a Calorhythm Slim AM-122 device (Tanita) for 7 days prior to the intervention and during the final 7 days of the intervention. Subjects were followed up between June 27, 2022 and November 25, 2022.

### Statistical analysis

The Shapiro–Wilk test was used to examine the normality of the background information for continuous data, and the F-test was performed for variance, followed by the Student’s t-test (with equal variances) or the Aspin–Welch’s t-test (without equal variances). Mann–Whitney U-test was also applied if normality was not demonstrated. χ^2^ tests were performed for nominal data.

Analysis of covariance was performed to control for the effects of confounders related to the primary and secondary outcomes. As a precondition analysis, the significance of the regression analysis for covariates and dependent variables and the parallelism between regression lines were determined, and covariance analysis was conducted using age [18–20] or pre-intervention values, or both, as covariates. In case the preconditions were not met, the same procedure used for the analysis of background information was used to test for differences in means and representative values.

Additionally, subgroup analyses were performed for walking speed, grip strength, and skeletal muscle mass based on gender, pre-intervention walking speed (≥ 1.0 m/s, < 1.0 m/s), J-CHS criteria (1–2 items denotes pre-frail, 3 items or more denotes frail), pre-intervention average step counts (≥ 5,000 steps/day, < 5,000 steps/day), pre-intervention total energy consumption (2,100 kcal for men and 1,650 kcal for women, as boundary conditions). The criterion of steps was determined in a previous study on dementia prevention [21], and the total energy consumption was set using the estimated energy requirements depending on activity level, as described in the Dietary Reference Intakes for Japanese [22].

As a supplementary analysis, for subgroups based on pre-intervention walking speed (≥ 1.0 m/s, < 1.0 m/s) and pre-intervention number of steps (≥ 5,000 steps/day, < 5,000 steps/day), the number of applicable pre-intervention J-CHS criteria items (physical activity, fatigue, weight loss, walking speed, and grip strength) was tabulated and compared between groups using the χ^2^ test.

IBM SPSS Statistics v25 and R v3.6.3 (02-29-2020) were used to perform the analyses, and a two-tailed test with a significance level of less than 5% was considered significantly different.

### Ethics approval statement

This study was approved by the Healthcare Systems Ethics Committee (approval number: 2142) in accordance with the ethical principles outlined in the Declaration of Helsinki and the Ethical Guidelines for Life Sciences and Medical Research Involving Human Subjects. The trial was registered in the UMIN Clinical Trials Registry (UMIN000044999).

## Results

**Fig. 1:**
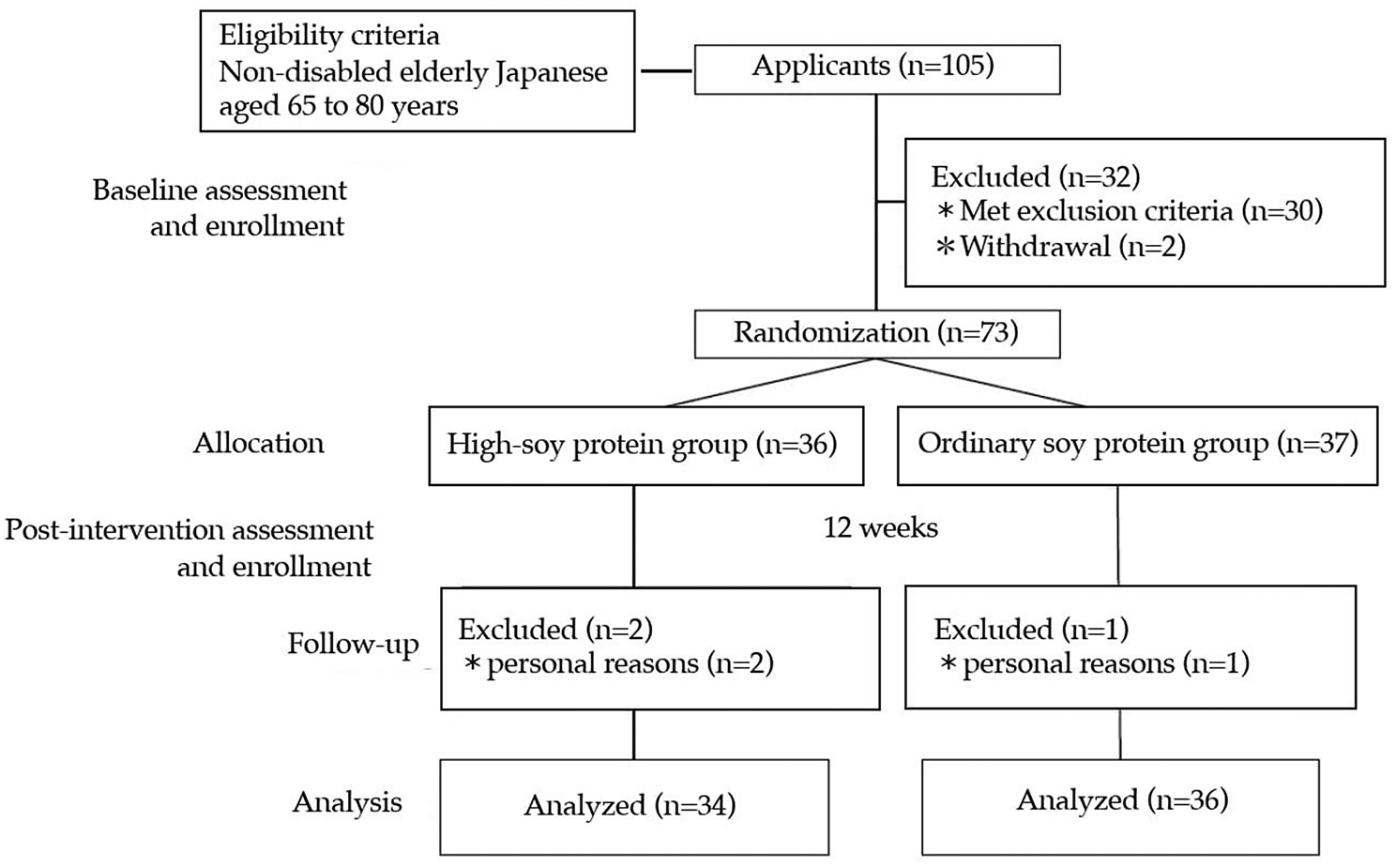
Flow chart of subject participation in the study

Figure 1 shows that 70 of the 73 subjects were included in the final analysis, excluding 3 subjects who dropped out for personal reasons.

The average age of the subjects in the final analysis was 72.0 ± 4.9 years in the high-soy protein group and 71.6 ± 5.1 years in the control group. There were no significant differences in background data between the two groups, as shown in Table 1.

**Table 1:**
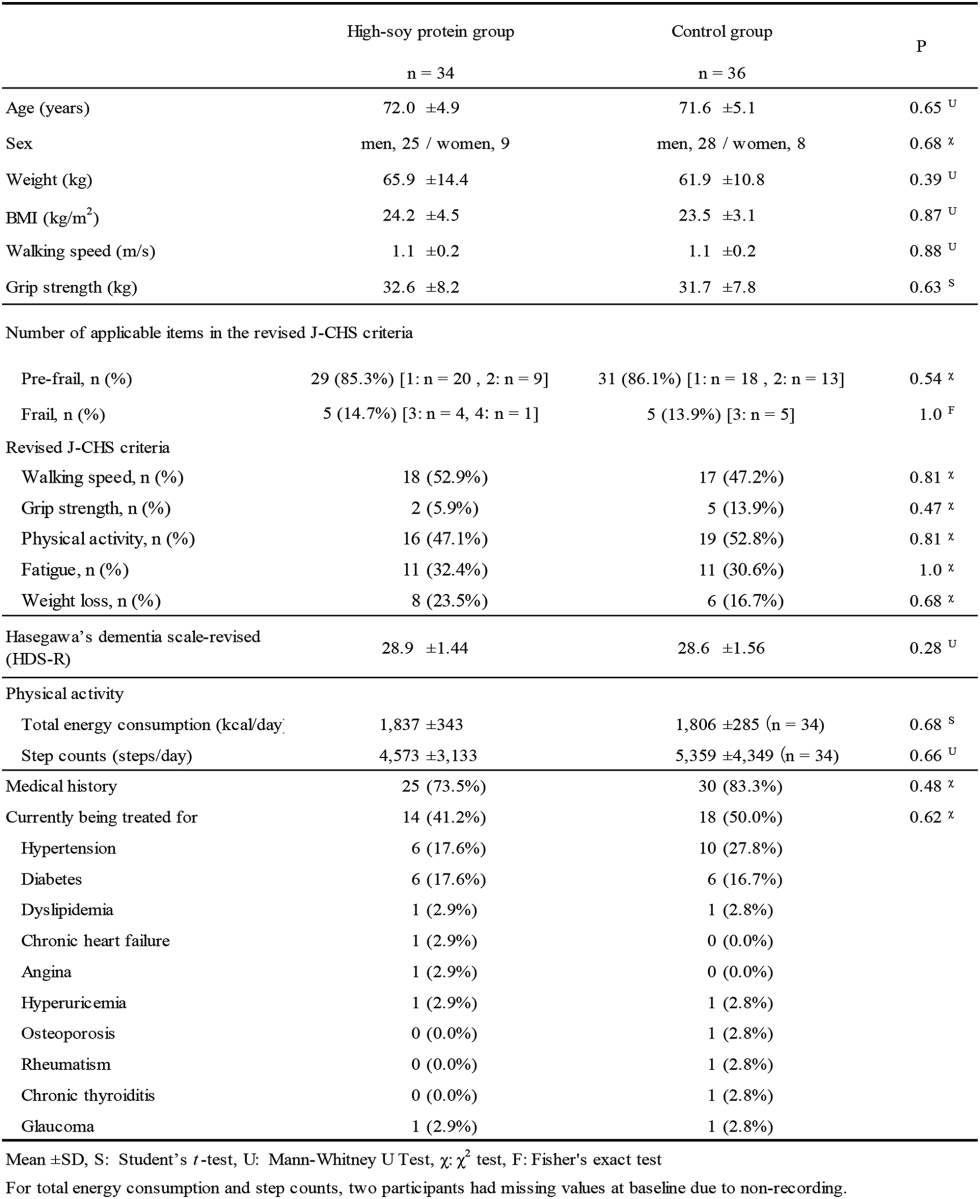
Baseline characteristics of elderly participants

**Fig. 2:**
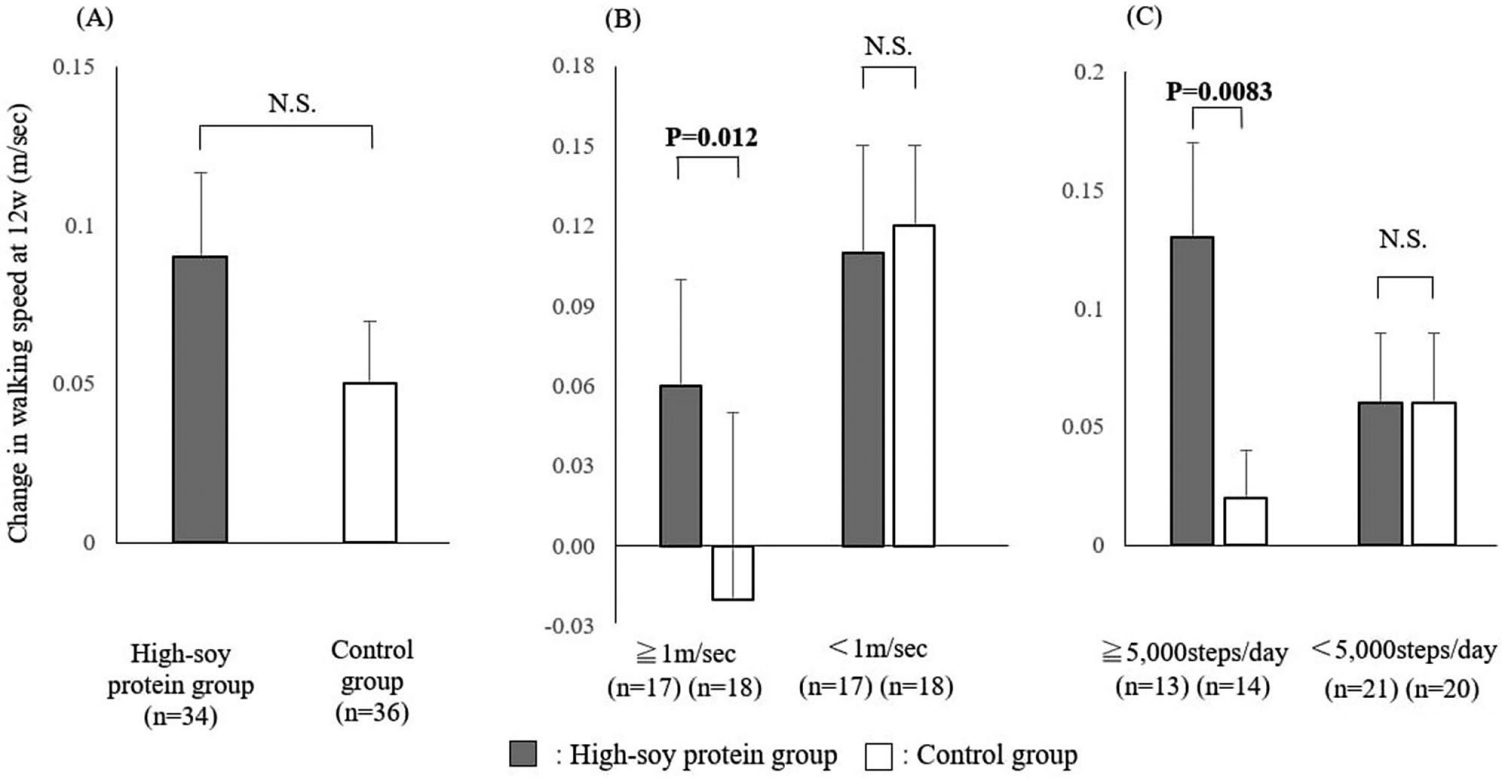
Changes in walking speed and step counts during the 12-week intervention. (**A**) Analysis of all subjects, (**B**) subgroup analysis of walking speed, and (**C**) subgroup analysis of step counts. Closed and open bars indicate the values for the high-soy protein and control groups, respectively

Notably, the change in walking speed after 12 weeks of intervention in the high-soy protein group was increased, but no significant difference was observed between the two groups. In the subgroup with a pre-intervention walking speed of 1.0 m/s or greater (*n* = 35), a significant increase in walking speed was observed in the high-soy protein group (*n* = 17) compared with the control group (*n* = 18) (*P* = 0.012), as shown in Table 2; Fig. 2.

**Table 2:** Changes in walking speed, grip strength, and skeletal muscle mass

		Walking speed (m/s)					Grip strength (kg)					Skeletal muscle mass (kg)				
		n	Baseline	Week 12	Change (95% CI)	P	n	Baseline	Week 12	Change (95% CI)	P	n	Baseline	Week 12	Change (95% CI)	P
All analysis subjects	High-soy protein group	34	1.06 ± 0.03	1.15 ± 0.04	0.09 ± 0.03 (0.03,0.14)	0.26^b^	34	32.6 ± 1.4	32.4 ± 1.4	-0.2 ± 0.4 (-1.0, 0.6)	0.12^S^	34	25.4 ± 0.9	25.4 ± 0.9	0.0 ± 0.1 (-0.1, 0.2)	0.91^S^
	Control group	36	1.07 ± 0.03	1.11 ± 0.02	0.05 ± 0.02 (0.01,0.09)		36	31.7 ± 1.3	32.4 ± 1.4	0.8 ± 0.5(-0.2,1.8)		34^††^	24.2 ± 0.7	24.3 ± 0.7	0.1 ± 0.1(-0.1,0.2)	
Subgroup analyses																
Men	High-soy protein group	25	1.00 ± 0.04	1.11 ± 0.05	0.11 ± 0.03(0.04,0.18)	0.09^b^	25	36.1 ± 1.3	35.8 ± 1.1	-0.3 ± 0.5(-1.2,0.7)	0.13^S^	25	27.5 ± 0.9	27.5 ± 0.9	0.0 ± 0.1(-0.2,0.3	0.93^S^
	Control group	28	1.07 ± 0.04	1.10 ± 0.03	0.03 ± 0.02(-0.01,0.07)		28	34.5 ± 1.2	35.4 ± 1.3	0.9 ± 0.6(-0.3,2.2)		26^††^	25.9 ± 0.6	25.9 ± 0.5	0.1 ± 0.1(-0.2,0.3)	
Women	High-soy protein group	9	1.22 ± 0.05	1.25 ± 0.05	0.03 ± 0.03 (-0.05, 0.10)	0.89^a^	9	22.8 ± 1.1	22.7 ± 1.5	-0.1 ± 0.7 (-1.6, 1.5)	0.77^S^	9	19.6 ± 0.6	19.7 ± 0.7	0.1 ± 0.2(-0.4,0.5)	0.94^S^
	Control group	8	1.04 ± 0.04	1.17 ± 0.04	0.13 ± 0.05 (0.00, 0.25)		8	21.9 ± 1.1	22.1 ± 1.0	0.2 ± 0.8 (-1.6, 2.0)		8	18.7 ± 0.9	18.8 ± 1.0	0.1 ± 0.2(-0.4,0.6)	
Walking speed: higher than 1.0 m/s (Baseline)	High-soy protein group	17	1.22 ± 0.03	1.28 ± 0.04	0.06 ± 0.04 (-0.01, 0.14)	0.012^b^	17	30.4 ± 2.0	30.3 ± 2.0	-0.1 ± 0.6 (-1.3, 1.2)	0.78^S^	17	23.2 ± 1.0	23.3 ± 1.0	0.1 ± 0.1(-0.2,0.4)	0.81^S^
	Control group	18	1.19 ± 0.04	1.17 ± 0.03	-0.02 ± 0.02 (-0.05, 0.02)		18	32.5 ± 2.2	32.2 ± 2.3	-0.3 ± 0.6 (-1.6, 1.0)		17^†^	23.3 ± 1.1	23.4 ± 1.1	0.1 ± 0.1(-0.1,0.3)	
Walking speed: less than 1.0 m/s (Baseline)	High-soy protein group	17	0.90 ± 0.02	1.01 ± 0.05	0.11 ± 0.04 (0.02, 0.19)	0.85^S^	17	34.8 ± 1.8	34.4 ± 1.8	-0.3 ± 0.5 (-1.4, 0.7)	0.016^S^	17	27.6 ± 1.3	27.6 ± 1.3	0.0 ± 0.1 (-0.3, 0.2)	0.72^S^
	Control group	18	0.94 ± 0.01	1.06 ± 0.03	0.12 ± 0.03 (0.06, 0.18)		18	30.8 ± 1.5	32.7 ± 1.6	1.9 ± 0.7 (0.4, 3.3)		17^†^	25.1 ± 0.9	25.1 ± 0.9	0.0 ± 0.2 (-0.3, 0.4)	
Frailty: At least 3 items meet the revised J-CHS criteria	High-soy protein group	5	0.91 ± 0.04	0.95 ± 0.14	0.04 ± 0.10 (-0.25, 0.32)	0.71^S^	5	33.5 ± 3.1	33.2 ± 3.1	-0.3 ± 0.7 (-2.1, 1.5)	0.76^S^	5	28.7 ± 3.6	28.4 ± 3.6	-0.3 ± 0.2 (-0.7, 0.1)	0.63^S^
	Control group	5	0.97 ± 0.06	1.05 ± 0.02	0.08 ± 0.05 (-0.07, 0.23)		5	27.6 ± 3.1	27.9 ± 4.1	0.3 ± 1.9 (-4.9, 5.6)		5	23.7 ± 0.8	23.5 ± 0.9	-0.2 ± 0.2 (-0.7, 0.3)	
Frailty: 1 or 2 items meet the revised J-CHS criteria	High-soy protein group	29	1.08 ± 0.04	1.18 ± 0.04	0.09 ± 0.03 (0.04, 0.15)	0.09^b^	29	32.4 ± 1.6	32.2 ± 1.5	-0.2 ± 0.4 (-1.1, 0.7)	0.13^S^	29	24.8 ± 0.8	24.9 ± 0.8	0.1 ± 0.1 (-0.1, 0.3)	0.98^S^
	Control group	31	1.08 ± 0.03	1.12 ± 0.03	0.04 ± 0.02 (0.00, 0.09)		31	32.3 ± 1.4	33.2 ± 1.5	0.9 ± 0.5 (-0.2, 1.9)		29^††^	24.3 ± 0.8	24.4 ± 0.8	0.1 ± 0.1 (-0.1, 0.3)	
Step counts: higher than 5,000 steps/day (Baseline)	High-soy protein group	13	1.11 ± 0.06	1.24 ± 0.06	0.13 ± 0.04 (0.05, 0.22)	0.0083^b^	13	35.3 ± 2.5	35.3 ± 2.2	0.0 ± 0.7 (-1.5, 1.5)	0.77^S^	13	26.1 ± 1.2	26.2 ± 1.2	0.2 ± 0.1 (-0.1, 0.4)	0.38^S^
	Control group	14	1.12 ± 0.04	1.14 ± 0.03	0.02 ± 0.02 (-0.03, 0.07)		14	32.0 ± 2.6	32.4 ± 2.7	0.3 ± 0.9 (-1.6, 2.2)		14	24.0 ± 1.2	24.0 ± 1.3	0.0 ± 0.1 (-0.2, 0.3)	
Step counts: less than 5,000 steps/day (Baseline)	High-soy protein group	21	1.03 ± 0.04	1.08 ± 0.05	0.06 ± 0.03 (-0.02, 0.13)	0.81^b^	21	30.9 ± 1.6	30.5 ± 1.6	-0.3 ± 0.5 (-1.3, 0.6)	0.10^S^	21	25.0 ± 1.3	24.9 ± 1.3	0.0 ± 0.1 (-0.3, 0.3)	0.46^S^
	Control group	20	1.04 ± 0.04	1.10 ± 0.03	0.06 ± 0.03 (0.00, 0.13)		20	31.8 ± 1.5	32.7 ± 1.5	1.0 ± 0.6(-0.3,2.2)		18^††^	24.6 ± 0.8	24.7 ± 0.8	0.1 ± 0.1 (-0.2, 0.4)	
High total energy consumption (Baseline)	High-soy protein group	7	1.05 ± 0.07	1.23 ± 0.05	0.18 ± 0.05 (0.05, 0.31)	0.52^b^	7	35.0 ± 3.1	35.2 ± 3.0	0.2 ± 1.1 (-2.6, 2.9)	0.22^S^	7	29.8 ± 2.7	29.9 ± 2.6	0.1 ± 0.2 (-0.3, 0.5)	0.73^S^
	Control group	8	1.04 ± 0.08	1.19 ± 0.05	0.15 ± 0.06 (0.01, 0.28)		8	31.5 ± 3.9	33.5 ± 3.7	2.0 ± 0.9 (-0.1, 4.1)		8	25.8 ± 1.7	25.8 ± 1.7	0.0 ± 0.3 (-0.6, 0.6)	
Men: Higher than 2,100 kcal/day																
Women: Higher than 1,650 kcal/day																
Low total energy consumption	High-soy protein group	27	1.06 ± 0.04	1.12 ± 0.05	0.06 ± 0.03 (0.00, 0.12)	0.16^W^	27	31.9 ± 1.6	31.6 ± 1.5	-0.3 ± 0.4 (-1.1, 0.5)	0.40^S^	27	24.3 ± 0.8	24.3 ± 0.8	0.0 ± 0.1 (-0.2, 0.3)	0.62^S^
(Baseline)	Control group	26	1.08 ± 0.03	1.09 ± 0.03	0.01 ± 0.02 (-0.02, 0.05)		26	32.0 ± 1.3	32.3 ± 1.5	0.3 ± 0.6 (-0.9, 1.5)		24^††^	23.8 ± 0.8	23.9 ± 0.8	0.1 ± 0.1 (-0.1, 0.3)	
Men: Less than 2,100 kcal/day																
Women: Less than 1,650 kcal/day																

Mean ± SE. P values were calculated using analysis of covariance (ANCOVA) adjusted for baseline value and age. Covariates are ^a^baseline value and age, ^b^baseline value. ^S^Student’s t-test. ^W^Aspin-Welch’s t-test.

For total energy consumption, two participants had missing values at baseline due to non-recording

^†^ Data missing for one participant

^††^Data missing for two participants

Furthermore, in the subgroup with more than 5,000 steps/day prior to intervention (*n* = 27), the improvement in walking speed was significantly higher (*P* = 0.0083) in the high-soy protein group (*n* = 13) than in the control group (*n* = 14). In contrast, we found no significant difference in the grip strength or skeletal mass after 12 weeks of intervention between the two groups (including the subgroup analyses).

Next, we divided the J-CHS questionnaire items into three groups (applicable to not applicable, no change, and applicable to not applicable) based on the number of applicable or not applicable before and after the 12-week intervention, and compared the number ratios between groups. As shown in Table 3, no significant differences in physical activity or fatigue were observed between the two groups (*P* = 0.40, 0.85).

**Table 3:** Changes in the number of applicable items based on the revised J-CHS criteria

		*n*	0: Not Applicable, 1: Applicable				Change			P
			Baseline		Week 12					
			0	1	0	1	-1	0	1	
Physical activity	High-soy protein group	34	18	16	17	17	4	27	3	0.40^χ^
	Control group	36	17	19	17	19	5	24	7	
Fatigue	High-soy protein group	34	23	11	24	10	6	22	6	0.85^χ^
	Control group	36	25	11	25	11	8	21	7	

Applicable criteria (Physical activity): Subjects answered “not more than once a week” to question 1) “Do you perform light exercise/exercise?” and question 2) “Do you perform regular exercise/sports?”

Applicable criteria (Fatigue): Subjects answered “Yes” to question 3) “Have you been feeling inexplicably tired for the past two weeks?”

Change: -1) Applicable to not applicable, 0) No change, 1) Not applicable to applicable.

χ: χ2 test.

In addition, as shown in Table 4, comparing the number of pre-intervention applicable J-CHS items, there were no significant differences between subgroups based on the pre-intervention number of steps (≥ 5,000 steps/day, < 5,000 steps/day) for any of the items. For subgroups based on walking speed (≥ 1.0 m/s, < 1.0 m/s), there were no significant differences between groups for any of the items, except for the walking speed.

**Table 4:**
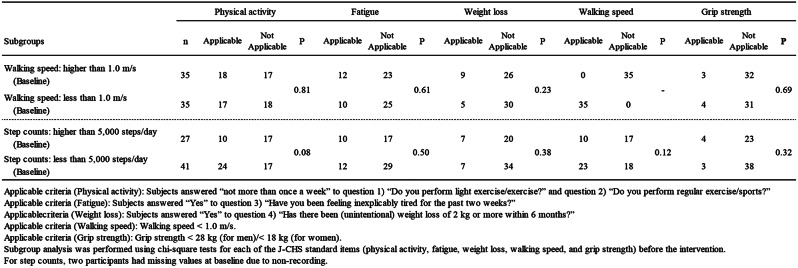
Distribution of the number of applicable items based on the revised J-CHS criteria among subgroups

We also observed no significant differences in other physical activities, such as the number of steps or total consumption of energy between groups, either before or after the 12-week intervention, as shown in Table 5.

**Table 5:**
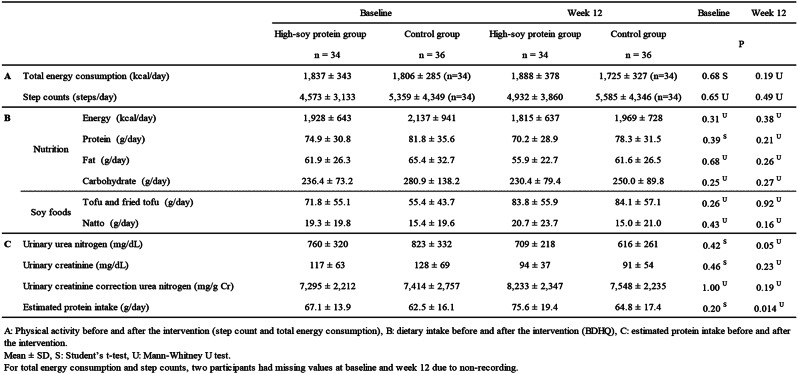
Physical activity, dietary intake, and estimated protein intake before and after the intervention

Then, energy intake and carbohydrate, fat, and protein intake were investigated using the BDHQ. Table 5 shows that no significant differences were observed between the high-soy protein and control groups either before or after the intervention. Additionally, we found no significant differences in the intake of soy foods other than soymilk (e.g., tofu and fried tofu) between the two groups, as shown in Table 5.

Finally, the protein intake estimated from urinary urea nitrogen showed no significant differences between the two groups prior to the intervention. However, the high-soy protein group exhibited significantly higher protein intake than the control group after the intervention.

## Discussion

Our study on the effects of ingesting soy milk high in soy protein (14.5 g soy protein/day) for 12 weeks (without additional exercise intervention) provided valuable insight into nutritional intervention and physical frailty. Concerning the overall cohort, no significant improvement in frailty was observed for the high-soy protein or the control groups; however, subgroup analyses revealed a significant improvement in walking speed among subjects with a pre-intervention walking speed and footstep count of at least 1.0 m/s and 5,000 steps/day, respectively.

The minimal clinically important difference in walking speed among community-dwelling elderly people is reported to be 0.05 m/s (0.04–0.06 m/s). Therefore, the change in walking speed in the high-soy protein group (0.06 ± 0.15 m/s) and the difference between groups (0.08 m/s) are considered to be clinically significant [23].

This suggests that soy protein intake by soymilk ingestion supports an improvement in locomotor function among the Japanese frail and pre-frail elderly population with relatively high levels of routine locomotor function. Meanwhile, a previous study showed no improvement in locomotor function with nutritional intervention by ingestion of a beverage containing 15 g of soy protein for 10 weeks alone in frail elderly residents at a British institution [10]. The outcomes of the present study demonstrate the feasibility of improving frailty through soymilk intake, but the results represent an exploratory subgroup analysis, and further validation is required in future studies.

The improvement in walking speed with soymilk ingestion may be attributed to the promotion of muscle synthesis or the suppression of muscle atrophy by soy protein. However, in this study, there was no significant improvement in grip strength or skeletal muscle mass in either the overall analysis or the subgroup analysis. Concerning grip strength and skeletal muscle mass, nutritional intervention alone in the frail elderly rarely results in improvement, and additional exercise intervention appears to be necessary [9, 24]. It was previously shown that muscle strength can be improved by nutritional intervention alone; however, the amount of protein in the intervention (20–40 g/day) was higher than that in our study [11]. Therefore, we infer that the implementation of moderate exercise intervention or increased protein intake is necessary to improve grip strength and skeletal muscle mass.

Moreover, in addition to soy protein, soymilk contains isoflavones, which are known to possess physiological functions for muscle [25]. The extent to which the individual effects and synergistic effects of soy protein and isoflavones influenced the results of this study remains unclear. Nevertheless, in the present RCT using different soy protein contents, it is improbable that there was any significant isoflavone-derived effect, which was similarly present in the control beverage.

From these results, although there was no increase in exercise or step count between the two groups, the subgroups with a higher walking speed (≥ 1.0 m/s) or higher step count (≥ 5,000 steps/day) exercised or played sports as part of their daily physical activities, which may be one of the reasons for the improvement in walking speed. However, referring to the result of J-CHS questionnaire items (especially fatigue and physical activity), no significant difference was seen between the high-soy protein group and the control group through the intervention, strongly suggesting that there were no drastic changes in exercise habits. Furthermore, the subgroup analysis of the efficacy outcome items related to energy consumption prior to the intervention revealed no significant change in exercise function independent of energy consumption.

These results indicate that the influence of individual differences in daily exercise habits and activity levels was low. Additionally, the BDHQ confirmed no significant differences in the intake of major nutrients or soy foods, except soymilk (tofu and fried tofu), between the two groups before or after the intervention. Notably, the estimated protein intake based on urine analysis was higher in the high-soy protein group than in the control group after 12 weeks of intervention. Therefore, the effect of nutrient intake from dietary habits other than nutritional intervention was considered to be small.

## Conclusion

An RCT was conducted to examine the impact of a 12-week intervention using soymilk high in soy protein (14.5 g soy protein/200 ml) on frailty in the Japanese pre-frail or frail elderly aged 65 to 85 years. The results of an exploratory subgroup analysis suggested that continued intake of soy milk with high soy protein content improves walking speed for the elderly with an ordinary walking speed of at least 1.0 m/s and a step count of at least 5,000 steps/day.

## Data Availability

The data that support the findings of this study are available on request from the corresponding author. The data are not publicly available due to ethical restrictions.
